# One-year outcomes of polymer-free amphilimus-eluting stents versus durable polymer zotarolimus-eluting stents in patients with diabetes mellitus: a meta-analysis

**DOI:** 10.1186/s12933-022-01673-8

**Published:** 2022-10-28

**Authors:** Hong Wang, Xiaoya Xie, Quannan Zu, Ming Lu, Rongfa Chen, Zhiren Yang, Yongqiang Gao

**Affiliations:** 1 Department of Cardiology, The People’s Hospital of Guangxi Zhuang Autonomous Region, Nanning, 530021 Guangxi People’s Republic of China; 2 Macau University of Science and Technology, Macau, People’s Republic of China; 3 College of Management and Economics, Tianjin University, Tianjin, 300072 People’s Republic of China; 4 The State Key Laboratory Management and Control for Complex Systems, Institute of Automation, Chinese Academy of Sciences, Beijing, 100190 People’s Republic of China

**Keywords:** Diabetes mellitus, Polymer-free Amphilimus-eluting stents, Durable polymer zotarolimus-eluting stents, Percutaneous coronary intervention, Cardiovascular outcomes, Target lesion failure, Major adverse cardiac events, Stent thrombosis

## Abstract

**Background:**

Diabetes mellitus (DM) and cardiovascular diseases often co-exist. Today, percutaneous coronary intervention (PCI) is the preferred revascularization procedure for majority of patients with coronary artery disease. Polymer-free amphilimus-eluting stents (AES) represent a novel elution technology in the current era of drug-eluting stents. In this analysis, we aimed to systematically compare the cardiovascular outcomes which are associated with polymer-free amphilimus-eluting stents (AES) versus the durable polymer zotarolimus-eluting stents (ZES) for the treatment of patients with DM.

**Methods:**

Http://www.ClinicalTrials.gov, EMBASE, Web of Science, MEDLINE, Cochrane database and Google Scholar were searched for publications comparing polymer-free AES versus durable polymer ZES in patients with DM. Selective cardiovascular outcomes were assessed. Statistical analysis was carried out by the latest version of the RevMan software. Risk ratio (RR) with 95% confidence interval (CI) was used to represent the data analysis.

**Results:**

Four studies with a total number of 1795 participants with DM whereby 912 patients were assigned to be revascularized by the polymer-free AES and 883 patients were assigned to be revascularized by the durable polymer ZES were included in this analysis. In patients with DM, at one year, polymer-free AES were associated with significantly lower risk of major adverse cardiac events (MACEs) (RR: 0.69, 95% CI: 0.54–0.88; P = 0.002) and target lesion failure (TLF) (RR: 0.66, 95% CI: 0.48–0.91; P = 0.01) compared to durable polymer ZES. However, there was no significant change in all-cause mortality (RR: 0.79, 95% CI: 0.51–1.22; P = 0.28), cardiac death and the other cardiovascular outcomes. Similar risk of total stent thrombosis (RR: 1.13, 95% CI: 0.60–2.13; P = 0.70), including definite stent thrombosis (RR: 1.12, 95% CI: 0.38–3.31; P = 0.84), probable stent thrombosis (RR: 0.87, 95% CI: 0.37–2.09; P = 0.76), possible stent thrombosis (RR: 1.19, 95% CI: 0.50–2.87; P = 0.69) and late stent thrombosis (RR: 1.00, 95% CI: 0.17–5.72; P = 1.00) as between polymer-free AES and durable polymer ZES in patients with DM.

**Conclusions:**

At 1 year follow-up, polymer-free AES were associated with significantly lower MACEs and TLF compared to durable polymer ZES in these patients with DM, without any increase in mortality, stent thrombosis and other cardiovascular outcomes. However, this analysis is only based on a follow-up time period of one year, therefore, future research should focus on the long term follow-up time period.

## Background

Diabetes mellitus (DM) and cardiovascular diseases often co-exist [1]. Patients with DM are more prone to coronary artery disease due to endothelial dysfunction, vascular inflammation and thrombosis which later manifest as myocardial infarction and death [2]. Epidemiologic reports show the global prevalence of DM to rise with over 200 million people (expectation from year 2015 to 2040) [3]. Beside coronary artery disease, patients with DM are more susceptible to adverse cardiovascular outcomes following treatment interventions [4]. Hence, treating this subgroup of patients will become a necessity in the coming years due to an ageing population.

Today, percutaneous coronary intervention (PCI) is the preferred revascularization procedure for majority of the patients based on the number of coronary vessels which were involved, the extent of disease, the co-morbidities that exist, and any other medical condition or previous cardiovascular treatments [5].

Durable polymer everolimus-eluting stents (EES), and zotarolimus-eluting stents (ZES) are among the latest effective drug-eluting stents (DES) which have been used during revascularization of coronary arteries [6, 7]. Even though new-generation DES have been developed, DM has shown to often be an independent factor associated with adverse clinical outcomes following PCI [8].

Polymer-free amphilimus-eluting stents (AES) represent a novel elution technology in the current era of DES [9]. However, the clinical safety and efficacy of polymer-free AES as compared to the latest-generation permanent-polymer ZES have not yet been investigated in larger trials. Trials with a small number of participants, and other retrospective studies have recently investigated the benefits of polymer-free AES.

Patients with DM are most at risk for adverse events, especially ischemic events and stent thrombosis following PCI. It would therefore be interesting to compare a very effective durable polymer ZES with a newer polymer-free AES in patients with DM.

## Methods

### Search databases and searched strategies

Http://www.ClinicalTrials.gov, EMBASE, Web of Science, MEDLINE (including its subset PubMed), Cochrane database and Google Scholar were searched for publications comparing polymer-free AES versus durable polymer ZES in patients with DM.

The following search terms or phrases were used:

- Polymer-free Amphilimus-eluting stents versus durable polymer zotarolimus-eluting stents;
- Polymer-free Amphilimus-eluting stents versus durable polymer zotarolimus-eluting stents and diabetes mellitus;
- Amphilimus-eluting stents versus zotarolimus-eluting stents.

Abbreviations such as AES and ZES were also used

### Inclusion and exclusion criteria

Studies were included if:

- They compared polymer-free AES versus durable polymer ZES;
- They included patients with DM;
- They reported adverse cardiovascular outcomes as their endpoints;
- They were published in English;
- They were trials or observational studies.
  Studies were excluded if:
- They did not include patients with DM;
- They were systematic reviews, meta-analyses, literature reviews or case studies;
- They were duplicated studies.

### Definitions, outcomes and follow-up

The outcomes which were reported in the original studies have been listed in Table 1. Selective outcomes were assessed, and the endpoints of this analysis included:

- Major adverse cardiovascular events (MACEs) including a composite outcome of all-cause mortality, myocardial infarction, revascularization ± stroke;
- Target lesion failure (TLF) which was defined as a composite of cardiac death, target vessel myocardial infarction and target lesion revascularization;
- All-cause mortality;
- Cardiac death;
- Myocardial infarction (MI);
- Any revascularization;
- Target lesion revascularization (TLR);
- Target vessel revascularization (TVR);
- Total stent thrombosis;
- Definite stent thrombosis;
- Probable stent thrombosis;
- Possible stent thrombosis;
- Late stent thrombosis.

**Table 1:** Outcomes which were reported

Studies	Outcomes reported	Follow up time period
Hemert 2020 [13]	TLF, NACE, all-cause mortality, cardiac death, MI, stent thrombosis, late stent thrombosis, any unplanned revascularization, TLR, stroke, major bleeding	12 months
Romaguera 2022 [14]	TLF, cardiac death, MI, all-cause mortality, any revascularization, TVR, stent thrombosis, late stent thrombosis, acute and sub-acute stent thrombosis, definite, possible and probable stent thrombosis, major adverse cardiac events	12 months
Rozemeijer 2017 [15]	All-cause mortality, TLF, cardiac death, MI, TLR, TVR, any revascularization, stent thrombosis, definite, possible and probable stent thrombosis, early and late stent thrombosis, major adverse cardiac events, major bleeding (BARC ≥ 3)	12 months
Rozemeijer 2018 [16]	Major adverse cardiac events, TLF, all-cause death, cardiac death, MI, definite or probable stent thrombosis, stent thrombosis, stroke, TLR, major bleeding (BARC ≥ 3)	12 months

*NACE* Net adverse cardiovascular events, *TLF* target lesion failure, *MI* Myocardial infarction, *TLR* Target lesion revascularization, *TVR* Target vessel revascularization, *BARC* Bleeding defined by the academic research consortium

Outcomes such as acute and sub-acute stent thrombosis, bleeding outcomes and stroke were not assessed since they were reported in only one study, where a comparison would have been impossible.

This analysis had a follow-up time period of 12 months.

### Data extraction and quality assessment

The authors independently extracted data from the original studies. After carefully reading the selected studies, data including the authors’ names, year of publication, number of participants with DM who were assigned to polymer-free AES and durable polymer ZES, the type of studies, the baseline features of the participants, the year of participant enrollment, the endpoints which were reported, the total number of events associated with each endpoints were extracted.

During this data extraction process, any disagreement was resolved by carefully discussing the case with the corresponding author who was the one to make a final decision.

The methodological assessment was carried out by the Newcastle Ottawa Scale (NOS) [10] for observational studies, and the recommendations from the Cochrane collaboration [11] for the randomized trials. Grades were allotted signifying low, moderate or high risk of bias.

### Statistical analysis

Statistical analysis was carried out by the latest version of the RevMan software, RevMan 5.4. Heterogeneity was assessed by two simple statistical methods. First of all heterogeneity was assessed by the Q statistic test whereby an outcome with a P value less or equal to 0.05 was considered to be statistically significant, and an outcome with a P value above 0.05 was not considered to have reach statistical significance. Heterogeneity was also assessed by the I^2^ statistical test, whereby heterogeneity increased with an increasing I^2^ value. If the I^2^ value was less than 50%, a fixed statistical effect model was used, or else a random statistical effect model was used. Risk ratio (RR) with 95% confidence interval (CI) was used to represent the data analysis.

Sensitivity analysis was also carried out to rule out any influence of a specific study on the outcomes. Publication bias was also visually estimated through funnel plots.

### Compliance with ethical guidelines

This study is a meta-analysis, and does not involve experiment on humans or animals carried out by any of the authors. Hence, an ethical or board review approval was not required. Data were extracted from previously published original studies.

## Results

### Search outcomes

The preferred reporting items in systematic review and meta-analysis (PRISMA) guideline was followed [12]. A total number of 84 publications were obtained. Following a careful assessment of the titles and abstracts, 64 publications were eliminated due to irrelevance. Twenty (20) full text articles were assessed for eligibility. Further eliminations were carried out due to the following reasons:

- Studies were based on the same trial, that is, same data were used (4);
- Duplicated studies (12).

**Fig. 1:**
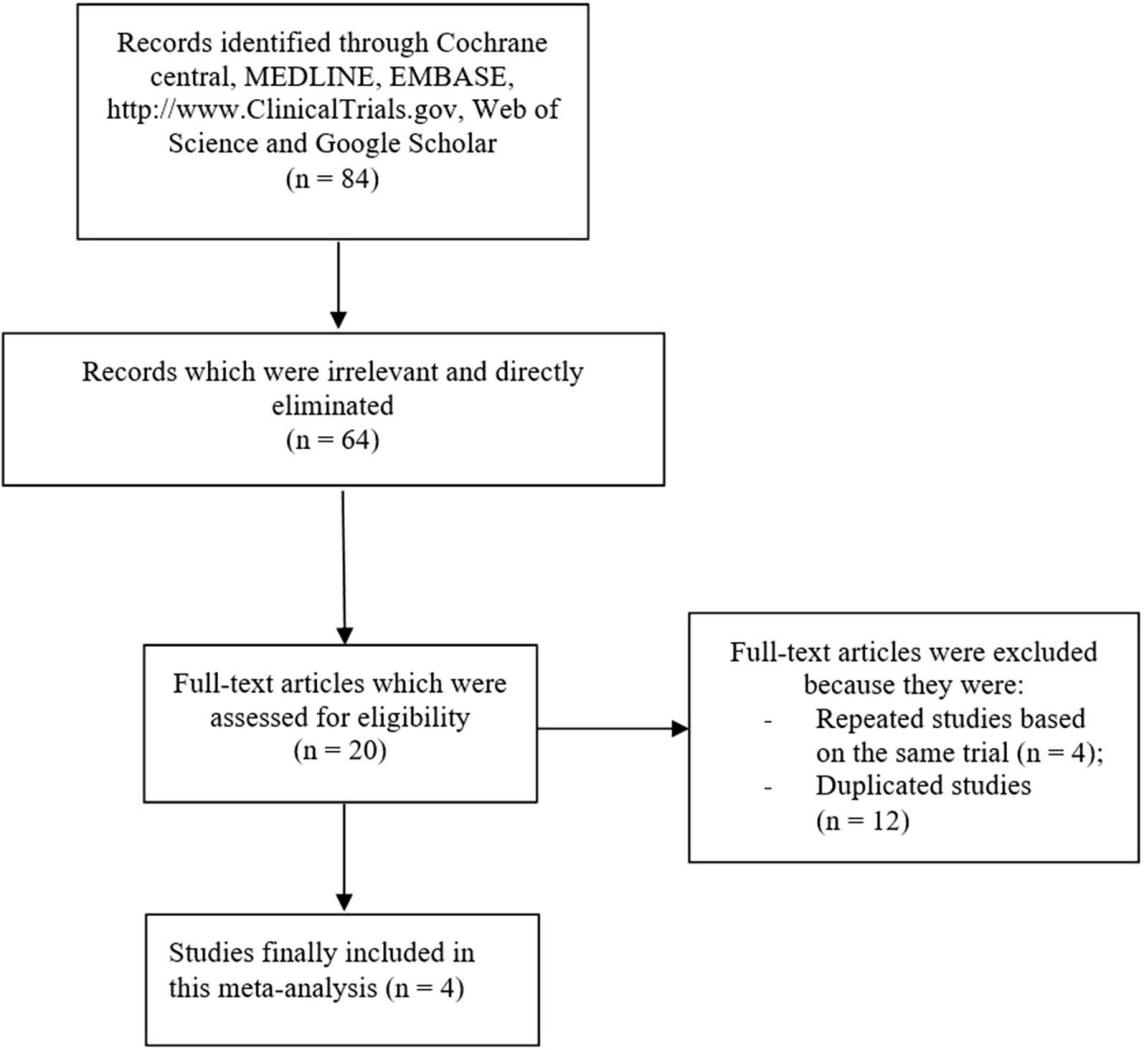
Flow diagram showing the study selection

Finally, only 4 studies [13–16] were selected for this analysis. The flow diagram for the study selection has been represented by Fig. 1.

### Main features of the studies

A total number of 1795 participants with DM were included in this analysis, whereby 912 patients were assigned to be revascularized by the polymer-free AES and 883 patients were assigned to be revascularized by the durable polymer ZES as shown in Table 2. This analysis included 4 studies whereby two studies were randomized trials and another two studies were observational studies. Based on the methodological assessment, a grade “B” was allotted to the studies implying moderate risk of bias.

**Table 2:** General features of the studies

Studies	No of patients with DM assigned to the polymer free amphilimus-eluting stents (n)	No of patients with DM assigned to the durable polymer zotarolimus eluting stents (n)	Type of study	Bias risk grade
Hemert 2020	150	153	Trial	B
Romaguera 2022	565	557	Trial	B
Rozemeijer 2017	85	80	Observational	B
Rozemeijer 2018	112	93	Observational	B
Total no of patients (n)	912	883		

*DM* Diabetes mellitus

### Baseline characteristics of the participants

The baseline characteristics of the DM participants were listed in Table 3. Majority of the participants were males (64.5% to 82.4%) with a mean age ranging from 65.9 to 69.2 years. Risk factors such as smoker (18.3% to 39.6%), hypertension (57.1% to 96.5%) and dyslipidemia (40.3% to 91.9%) were also listed in Table 3.

**Table 3:** Baseline features of the participants with diabetes mellitus

Studies	Mean age (years)	Males (%)	Smoker (%)	HTN (%)	DYS (%)
	AES/ZES	AES/ZES	AES/ZES	AES/ZES	AES/ZES
Hemert 2020	65.9/67.1	73.5/73.9	24.5/18.3	70.2/72.5	61.6/58.2
Romaguera 2022	68.6/67.2	76.6/74.5	18.9/24.4	84.1/82.9	82.8/80.0
Rozemeijer 2017	69.2/68.8	74.1/82.4	25.9/35.0	96.5/91.9	90.6/91.9
Rozemeijer 2018	66.5/66.8	72.9/64.5	34.3/39.6	59.6/57.1	42.0/40.3

*HTN* Hypertension, *DYS* Dyslipidemia, AES Polymer-free amphilimus-eluting stents, *ZES* Durable polymer zotarolimus eluting stents

### Main results of this analysis

**Fig. 2:**
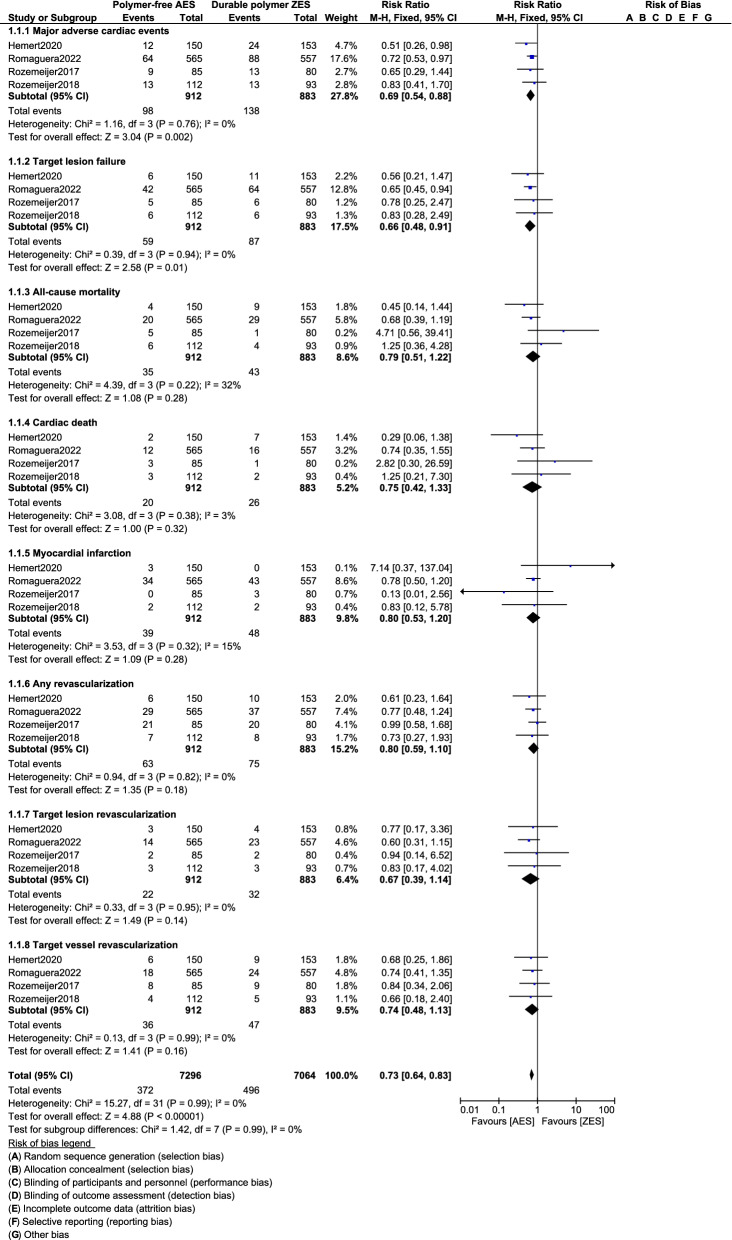
Cardiovascular outcomes observed with polymer-free AES versus durable polymer ZES in patients with DM

**Fig. 3:**
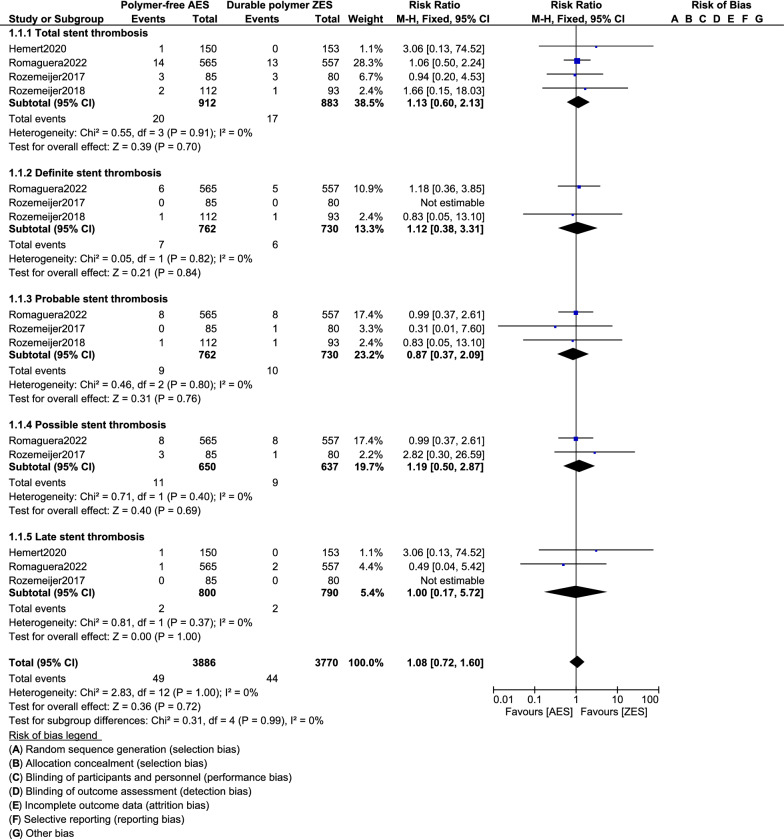
Stent thrombosis observed with polymer-free AES versus durable polymer ZES in patients with DM

Results of this analysis showed that in patients with DM, at one year, polymer-free AES were associated with significantly lower risk of MACEs (RR: 0.69, 95% CI: 0.54 – 0.88; P = 0.002) and TLF (RR: 0.66, 95% CI: 0.48–0.91; P = 0.01) compared to durable polymer ZES as shown in Fig. 2. However, there was no significant change in all-cause mortality (RR: 0.79, 95% CI: 0.51–1.22; P = 0.28), cardiac death (RR: 0.75, 95% CI: 0.42–1.33; P = 0.32), MI (RR: 0.80, 95% CI: 0.53–1.20; P = 0.28), any revascularization (RR: 0.80, 95% CI: 0.59–1.10; P = 0.18), TLR (RR: 0.67, 95% CI: 0.39–1.14; P = 0.14), and TVR (RR: 0.74, 95% CI: 0.48–1.13; P = 0.16) as shown in Fig. 2. Similar risk of total stent thrombosis (RR: 1.13, 95% CI: 0.60–2.13; P = 0.70), including definite stent thrombosis (RR: 1.12, 95% CI: 0.38–3.31; P = 0.84), probable stent thrombosis (RR: 0.87, 95% CI: 0.37–2.09; P = 0.76), possible stent thrombosis (RR: 1.19, 95% CI: 0.50–2.87; P = 0.69) and late stent thrombosis (RR: 1.00, 95% CI: 0.17–5.72; P = 1.00) as between polymer-free AES and durable polymer ZES in patients with DM as shown in Fig. 3.

**Fig. 4:**
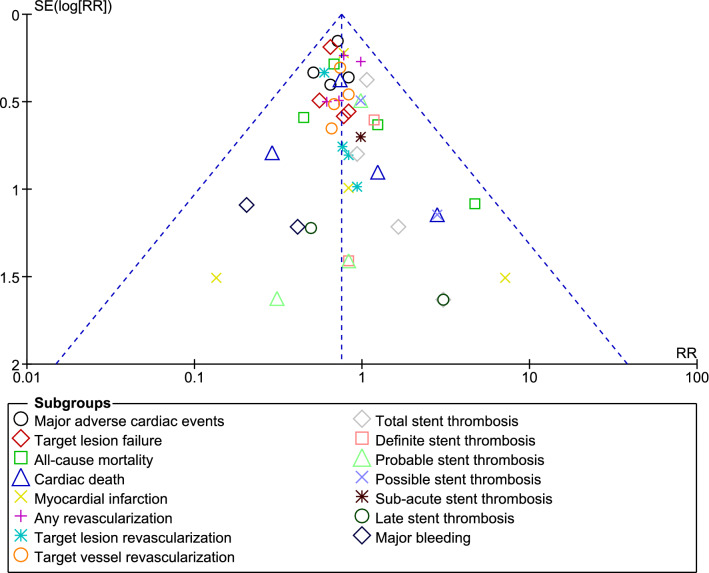
Funnel plot showing publication bias

Sensitivity analysis resulted in consistent results throughout. Publication bias was visually assessed through funnel plot. Based on this visual assessment, there was very little evidence of publication bias among the studies which were used to assess the clinical endpoints as shown in Fig. 4.

This comparison of outcomes between polymer-free AES versus durable polymer ZES in patients with DM has been summarized in Table 4.

**Table 4:** Results of this analysis

Outcomes which were assessed	RR with 95% CI	P value	I^2^ value (%)
Major adverse cardiac events	0.69 [0.54–0.88]	0.002	0
Target lesion failure	0.66 [0.48–0.91]	0.01	0
All-cause mortality	0.79 [0.51–1.22]	0.28	32
Cardiac death	0.75 [0.42–1.33]	0.32	3
Myocardial infarction	0.80 [0.53–1.20]	0.28	15
Any revascularization	0.80 [0.59–1.10]	0.18	0
Target lesion revascularization	0.67 [0.39–1.14]	0.14	0
Target vessel revascularization	0.74 [0.48–1.13]	0.16	0
Total stent thrombosis	1.13 [0.60–2.13]	0.70	0
Definite stent thrombosis	1.12 [0.38–3.31]	0.84	0
Probable stent thrombosis	0.87 [0.37–2.09]	0.76	0
Possible stent thrombosis	1.19 [0.50–2.87]	0.69	0
Late stent thrombosis	1.00 [0.17–5.72]	1.00	0

*RR* Risk ratio, *CI* confidence intervals

## Discussion

In this analysis, we compared the cardiovascular outcomes with polymer-free AES versus durable polymer ZES in patients with DM. The current results showed polymer-free AES to be associated with significantly lower TLF and MACEs in comparison to the durable polymer ZES in these patients with DM. Other outcomes including all-cause mortality, cardiac death, MI, TVR, TLR, and stent thrombosis were not significantly different.

Similarly, in the Randomized “All-comer” Evaluation of a Permanent Polymer Resolute Integrity Stent versus a Polymer FreeCre8 Stent (ReCre8) Landmark Analysis, a premiere in the head to head comparison comparing 3-year clinical outcomes after implantation of permanent-polymer versus polymer-free stents whereby a total number of 1491 participants were randomized and treated, the authors showed a similar rate of revascularization, mortality and stent thrombosis. In contrast, our results showed polymer-free AES to be associated with a significant decrease in the risk of TLF and MACEs [17]. It should be noted that our current study only involved participants with DM in contrast to the other study which included participants from the general population with coronary artery disease. A substudy of the ReCre8 trial consisting only of patients with DM showed composite of net adverse clinical events to be significantly higher with durable polymer ZES, and TLF was also higher with ZES among patients with insulin-treated DM [13].

Studies based on polymer-free AES versus durable polymer ZES in patients with DM were very limited. A randomized comparison of reservoir-based polymer-free AES versus durable polymer everolimus eluting stents (EES) in patients with diabetes mellitus (RESERVOIR trial) which was a multicenter, prospective, open-label, assessor-blinded, active treatment-controlled, randomized clinical trial and involving 112 participants with diabetes mellitus, with 40% insulin-treated patients, suggested a higher efficacy of AES which might be more beneficial in patients with DM [18].

New studies have shown chronic total occlusion to be more common among patients with DM. A multicenter experience based on the outcomes of the polymer-free AES for chronic total occlusion treatment and involving 235 participants demonstrated a lower rate of MACEs and target vessel failure for up to one year with the polymer-free stents supporting their use [19].

### Other factors to be considered

However, several other factors should be taken into consideration. A retrospective study involving 1574 patients with ACS undergoing coronary stenting showed that insulin resistance, represented by the triglyceride-glucose index, was independently and positively associated with DES in-stent restenosis [20]. This analysis was based on patients with diabetes mellitus, however, not only patients with diabetes mellitus but, patients with pre-diabetes were at a higher risk for ischemic events following newer generation stents implantation. This was shown in a post-hoc analysis whereby data from the BIO-RESORT and BIONYX stent trials were pooled for study analysis [21]. In addition, when second generation DES were compared with first generation DES, stent related adverse events were significantly lower with the second generation DES, however, it was observed that the use of dual antiplatelet therapy beyond one year did not reduce late stent thrombosis among patients who were revascularized with second generation DES, but late stent thrombosis was reduced in patients who were implanted with first generation DES [22]. It should also be noted that in a prospective study with over 7000 participants with diabetes mellitus, similar 3-year risk for TVF was observed with various types of contemporary DES [23].

### Novelty

At last, this manuscript is new in several ways. It is the first meta-analysis to assess the cardiovascular outcomes between polymer-free AES versus durable polymer ZES in patients with coronary artery disease with co-existing DM. Durable polymer ZES are still new drug eluting stents. A comparison between the most recent polymer-free AES versus the newer durable polymer ZES is a novelty in itself. This analysis has significant clinical implications in the way that better stents could be implanted in patients with DM, who are more at risk for thrombosis and ischemic events. By carrying out this research analysis, we expect to promote and encourage further research based on polymer-free AES and diabetes mellitus.

### Limitations

This study has certain limitations. First of all, we had a limited number of participants who were involved in the comparison of outcomes between polymer-free AES versus durable polymer ZES in these patients with DM, which could have an effect on the final outcomes. Another limitation might be the fact that several endpoints including stroke, acute and sub-acute stent thrombosis as well as bleeding events could not be assessed in this study because they were reported in only one original study, without data from other studies to compare. Moreover, even though cardiovascular drugs and extent of coronary occlusion and number of obstructed coronary arteries could also have had an impact on the final outcomes, they were not taken into consideration in this analysis. The duration of diabetes mellitus, and the number of participants on insulin therapy were ignored since several of the original studies did not report those details. At last, the permanent polymers ZES which were used were different in different studies. This could also have an impact on the results of this analysis.

## Conclusions

At 1 year follow-up, polymer-free AES were associated with significantly lower MACEs and TLF compared to durable polymer ZES in these patients with DM, without any increase in mortality, stent thrombosis and other cardiovascular outcomes. However, this analysis is only based on a follow-up time period of one year, therefore, future research should focus on the long term follow-up time period.

## Data Availability

All data and materials used in this research are freely available in electronic databases (MEDLINE, EMBASE, http://www.ClinicalTrials.gov, Web of Science, Cochrane database, Google scholar). References have been provided.

## Abbreviations

DM: Diabetes mellitus
AES: Amphilimus-eluting stents
ZES: Zotarolimus-eluting stents
PCI: Percutaneous coronary intervention
MACEs: Major adverse cardiac events
RR: Risk ratio
